# Kinetics of CD169, HLA-DR, and CD64 expression as predictive biomarkers of SARS-CoV2 outcome

**DOI:** 10.1186/s44158-023-00090-x

**Published:** 2023-03-27

**Authors:** Arianna Gatti, Paola Fassini, Antonino Mazzone, Stefano Rusconi, Bruno Brando, Giovanni Mistraletti

**Affiliations:** 1 Hematology Laboratory and Transfusion Center, New Hospital of Legnano: Ospedale Nuovo di Legnano, 20025 Legnano, Milano Italy; 2 Department of Intensive Care, New Hospital of Legnano: Ospedale Nuovo di Legnano, 20025 Legnano, Milano Italy; 3 Department of Internal Medicine, New Hospital of Legnano: Ospedale Nuovo di Legnano, 20025 Legnano, Milano Italy; 4 Department of Infectious Diseases, New Hospital of Legnano: Ospedale Nuovo di Legnano, 20025 Legnano, Milano Italy

**Keywords:** COVID-19, Monocyte CD169, Monocyte HLA-DR, Neutrophil CD64, Flow cytometry

## Abstract

**Introduction:**

Discriminating between virus-induced fever from superimposed bacterial infections is a common challenge in intensive care units. Superimposed bacterial infections can be detected in severe SARS-CoV2-infected patients, suggesting the important role of the bacteria in COVID-19 evolution. However, indicators of patients’ immune status may be of help in the management of critically ill subjects.

Monocyte CD169 is a type I interferon-inducible receptor that is up-regulated during viral infections, including COVID-19. Monocyte HLA-DR expression is an immunologic status marker, that decreases during immune exhaustion. This condition is an unfavorable prognostic biomarker in septic patients. Neutrophil CD64 upregulation is an established indicator of sepsis.

**Methods:**

In this study, we evaluated by flow cytometry the expression of cellular markers monocyte CD169, neutrophil CD64, and monocyte HLA-DR in 36 hospitalized patients with severe COVID-19, as possible indicators of ongoing progression of disease and of patients’ immune status. Blood testings started at ICU admission and were carried on throughout the ICU stay and extended in case of transfer to other units, when applicable.

The marker expression in mean fluorescence intensity (MFI) and their kinetics with time were correlated to the clinical outcome.

**Results:**

Patients with short hospital stay (≤15 days) and good outcome showed higher values of monocyte HLA-DR (median 17,478 MFI) than long hospital stay patients (>15 days, median 9590 MFI, *p*= 0.04) and than patients who died (median 5437 MFI, *p*= 0.05). In most cases, the recovery of the SARS-CoV2 infection-related signs was associated with the downregulation of monocyte CD169 within 17 days from disease onset. However in three surviving long hospital stay patients, a persistent upregulation of monocyte CD169 was observed. An increased neutrophil CD64 expression was found in two cases with a superimposed bacterial sepsis.

**Conclusion:**

Monocyte CD169, neutrophil CD64, and monocyte HLA-DR expression can be used as predictive biomarkers of SARS-CoV2 outcome in acutely infected patients. The combined analysis of these indicators can offer a real-time evaluation of patients’ immune status and of viral disease progression versus superimposed bacterial infections. This approach allows to better define the patients’ clinical status and outcome and may be useful to guide clinicians’ decisions. Our study focused on the discrimination between the activity of viral and bacterial infections and on the detection of the development of anergic states that may correlate with an unfavorable prognosis.

## Introduction

CD169 (Sialoadhesin or Siglec-1) is a type I interferon-inducible receptor, and as reported by several studies, its expression is upregulated on the surface of monocytes and dendritic cells during viral infections [1–3]. CD169 is normally expressed on resident macrophages, whereas it is virtually undetectable on quiescent monocytes in the peripheral blood of healthy subjects [4].

CD169 monocyte expression (*moCD169*) is increased upon the release of antiviral molecules, such as Interferon Type I [4]. It is also reported that moCD169 expression is increased in early SARS-CoV2 infection [5]. Among patients with mild COVID-19, a time-dependent expression of moCD169 with the highest values within the first 3 days after the onset of symptoms has been described, with expression levels returning to the normal range within the subsequent 3–4 weeks [6]. On the other hand, some studies showed a strong inflammatory response to SARS-CoV2 infection in severe cases, with also quantitative alterations of the monocyte and macrophage compartments [7, 8].

Monocytes/macrophages play a key role in the immune control of infections. Namely, monocyte HLA-DR (*moHLA-DR*) expression is an immunologic status marker, correlating with an efficient antigen-presenting function [9, 10]. Persistent over-stimulation or immune exhaustion induces the decrease of HLA-DR expression on monocytes, determining the drift to an anergic state. In several studies, the reduced moHLA-DR expression has been identified as an unfavorable prognostic biomarker during severe infections and sepsis [9, 11, 12]. Downregulation of moHLA-DR was also reported in critical COVID-19 patients, admitted to intensive care unit (ICU) [13, 14].

During prolonged stays in ICU for COVID-19, a common clinical problem is the discrimination between virus-induced findings and superimposed bacterial infections, which tend to occur in a later phase. The ordinary clinical parameters, i.e., fever, acute-phase reactant levels, lactate, blood, and respiratory tract cultures, do not always provide clues of the necessary sensitivity and specificity [15, 16]. It is well known that the high-affinity immunoglobulin Fc-gamma receptor type I CD64 is constitutively expressed at very low density on the surface of blood neutrophils in healthy subjects. The neutrophil CD64 density (*neCD64*) is promptly upregulated upon stimulation by bacteria and their soluble products, thus being considered a sensitive and specific marker of severe bacterial infections and sepsis [12, 17]. Sepsis is the most common cause of death among hospitalized patients in ICU [18]. The initial phase of sepsis is characterized by a hyperinflammatory status with an increase of pro-inflammatory markers (C-reactive protein, procalcitonin, IL- 6) and is followed by a immunosuppressive phase [19]. These markers are routinely used in the diagnosis and management of sepsis. As reported by several studies, these markers are also increased in patients with severe COVID-19, defining a condition known as “COVID-19 viral sepsis” [20, 21]. In addition, the co-occurrence of bacterial superinfections can be detected in severely SARS-CoV2 infected patients, suggesting the important role of bacteria in COVID-19 evolution [22, 23].

The aim of our study was therefore to evaluate by a quick, real-time flow cytometric assay if the combined expression of moCD169, moHLA-DR, and neCD64 in ICU patients with severe COVID-19 could allow a better definition of patients’ clinical status and outcome, to guide clinicians’ decisions. Our study focused on the discrimination between the activity of viral and bacterial infections and in the detection of developing anergic states, which may correlate with an unfavorable prognosis. The kinetics of moCD169, moHLA-DR, and neCD64 during the clinical course was also prospectively evaluated in some representative patients.

## Methods

### Patients and control group

Thirty-six hospitalized patients from December 2020 to April 2021 with severe COVID-19 were included in the study. Twenty-two patients (61%) were admitted to ICU and overall 6/36 (16.6%) died within a median of 44 days from admission. The characteristics of patients are reported in Table 1. Ten patients did not show any unfavorable risk factor at admission (i.e., diabetes, hypertension, obesity) nor major comorbidities (i.e., cardiovascular diseases, chronic obstructive pulmonary disease, or neoplasia) and one of them died. In five cases, only one risk factor was present. In other four patients, two unfavorable risk factors were present and two of them died. The remaining seventeen individuals showed at least three unfavorable comorbidities and three patients did not survive. Table 1 also summarizes the clinical severity scores (SOFa, SAPSII, P/F grade, P/F, and CCI) of all the patients enrolled in this study.

**Table 1:**
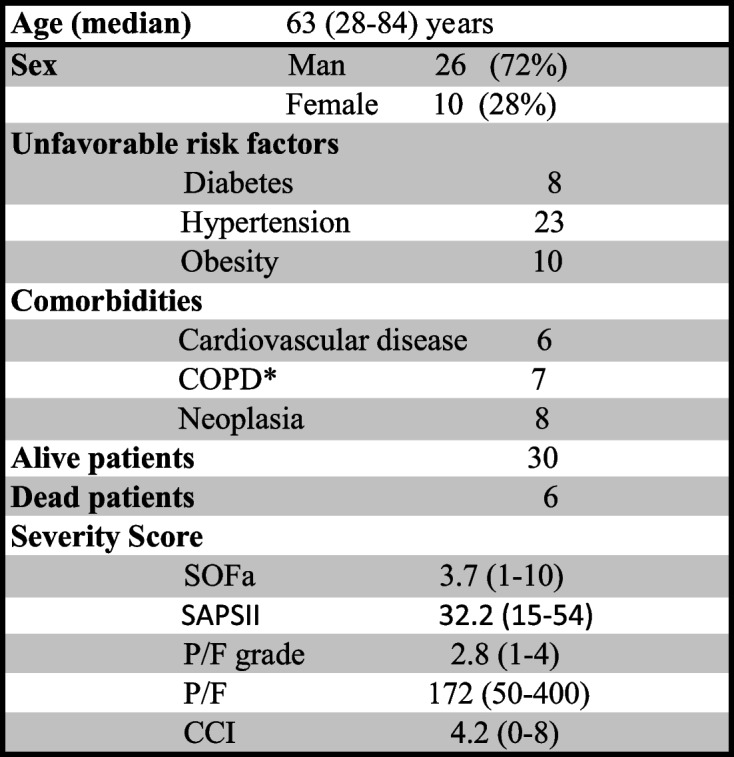
Characteristics of 36 patients included in the study

*COPD* chronic obstructive pulmonary disease

Ground-glass opacities at chest x-ray were present at admission in 64% of cases and in 8 patients pulmonary embolism was subsequently evidenced. In 33/36 cases, steroid treatment was undertaken in the early phase, along with support therapy. Blood testings started at hospital admission and were carried on throughout the ICU unit transfer and stay. In 15 subjects (42%), the prospective evaluation of moCD169, neCD64, and moHLA-DR during the clinical course was carried out, for a median of 12 days after the first analysis.

In addition, 10 age-matched healthy donors were included as the normal control group (NC).

The approval of this observational study was obtained from the local scientific committee, within the extended informed consent procedures activated during the early COVID-19 epidemic. The immunophenotypic testings were performed using the leftovers of routine full blood counts, and the study results were not used to undertake any clinical decision. In our institution, healthy blood donors sign informed consent for research studies on their routine samples, which are used to represent normal reference values in a variety of laboratory testings, including cell phenotyping.

### Flow cytometric analysis

Fifty microliters of EDTA-whole blood samples were taken from the fresh routine tubes ordered for the full blood count. Aliquots were mixed with 10 μl IOTest Myeloid Activation antibody cocktail (Beckman Coulter, Milano, Italy), containing anti-CD169-PE (R-Phycoerythrin, clone 7-239), anti-CD64-PB (Pacific Blue, clone 22), and anti-HLA-DR-APC (Allophycocyanin, clone Immu357) and incubated for 20 min at room temperature in the dark. The samples were processed with a lyse-no-wash technique using ammonium chloride and no fixation. Flow cytometric analysis was immediately performed on a FACS Lyric (Becton Dickinson-BD, Milano, Italy), daily calibrated using BD CS&T beads according to the recommended Euroflow procedures [24], with a specifically designed application setting and defined target values, following the manufacturer’s instructions. The samples were acquired immediately after the lysis at a medium rate.

Data from 100,000 total white cell events were acquired. Monocytes were identified with a Side Scatter (SSC)/CD64 dot plot. Granulocytes and lymphocytes were gated based on the respective SSC, CD64, and forward scatter (FSC) features (Fig. 4).

The degree of surface marker expression was quantitated by the mean fluorescence intensity (MFI) ratio. The MFI ratio is calculated by dividing the geometric mean of the positive fluorescence peak of the relevant population (i.e., moCD169) by the geometric mean of the negative control or reference population (i.e., lymphocytes) that is not expressing that marker.

Monocyte CD169 expression levels were calculated as the ratio between CD169 geometric mean fluorescence intensity (MFI) units on monocytes and MFI units on lymphocytes, which acted as the internal negative reference population.

CD64 expression level on granulocytes was similarly calculated as the ratio between CD64 MFI geometric mean units on granulocytes and CD64 MFI units on lymphocytes, which acted as the internal negative reference population.

MoHLA-DR expression level was calculated as plain geometric mean MFI, since no internal purely HLA-DR-negative reference cell populations can be found. Patient moHLA-DR is displayed in comparison to normal control subjects, as depicted in the examples in Fig. 5. The robustness and stability of MFI measurements over time were ensured by the daily calibration with CS&T beads.

### Statistical analysis

The distributions of surface antigen expression levels and ratios were checked for normality using the Kolmogorov-Smirnov test. In the case of non-normally distributed variates, statistical comparisons were performed using the non-parametric Mann-Whitney test. Comparisons between groups with normally distributed variates were performed using the Student *t*-test. Statistically significant differences were established by a *p* value < 0.05 in both cases.

## Results

The moCD169 MFI ratios and neCD64 MFI ratios were not normally distributed, as assessed with the Kolmogorov-Smirnov test. The Mann-Whitney test was therefore used for comparisons. MoHLA-DR expression levels showed a normal distribution both in patients and normal controls (NC), and Student’s *t*-test was used for comparisons.

**Fig. 1:**
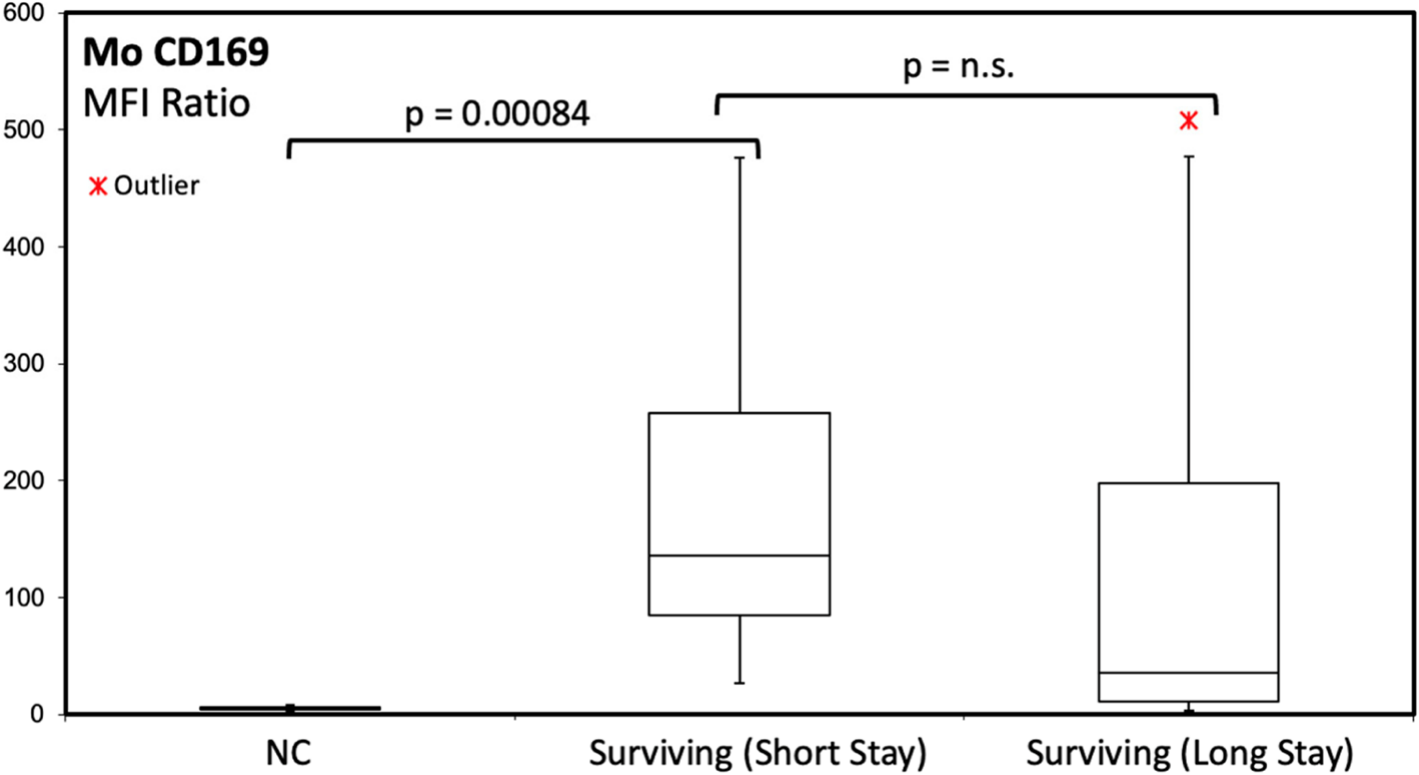
Expression of monocyte CD169 in acutely SARS-CoV2-infected patients in the early phase of the disease and during the follow-up, according to the length of hospital stay. The overall trend to moCD169 downregulation in the long-term did not reach statistical significance, due to some patients with persistent positive swabs for SARS-CoV2

SARS-CoV2-infected patients invariably expressed a strongly upregulated moCD169 during the early disease phase (Figs. 1 and 4) (mean 10 days from the beginning of symptoms, as reported at hospital admission by patients, where applicable, or by their relatives) as compared to quiescent monocytes in the NC group (mean moCD169 MFI ratio: 125 vs 5; *p*= 0.00084).

During the follow-up, moCD169 expression displayed a downward but individually variable trend, associated to viral clearance. We did not find any significant difference of expression of moCD169 between SARS-CoV2-infected patients admitted to ICU and those admitted to other clinical units.

**Fig. 2:**
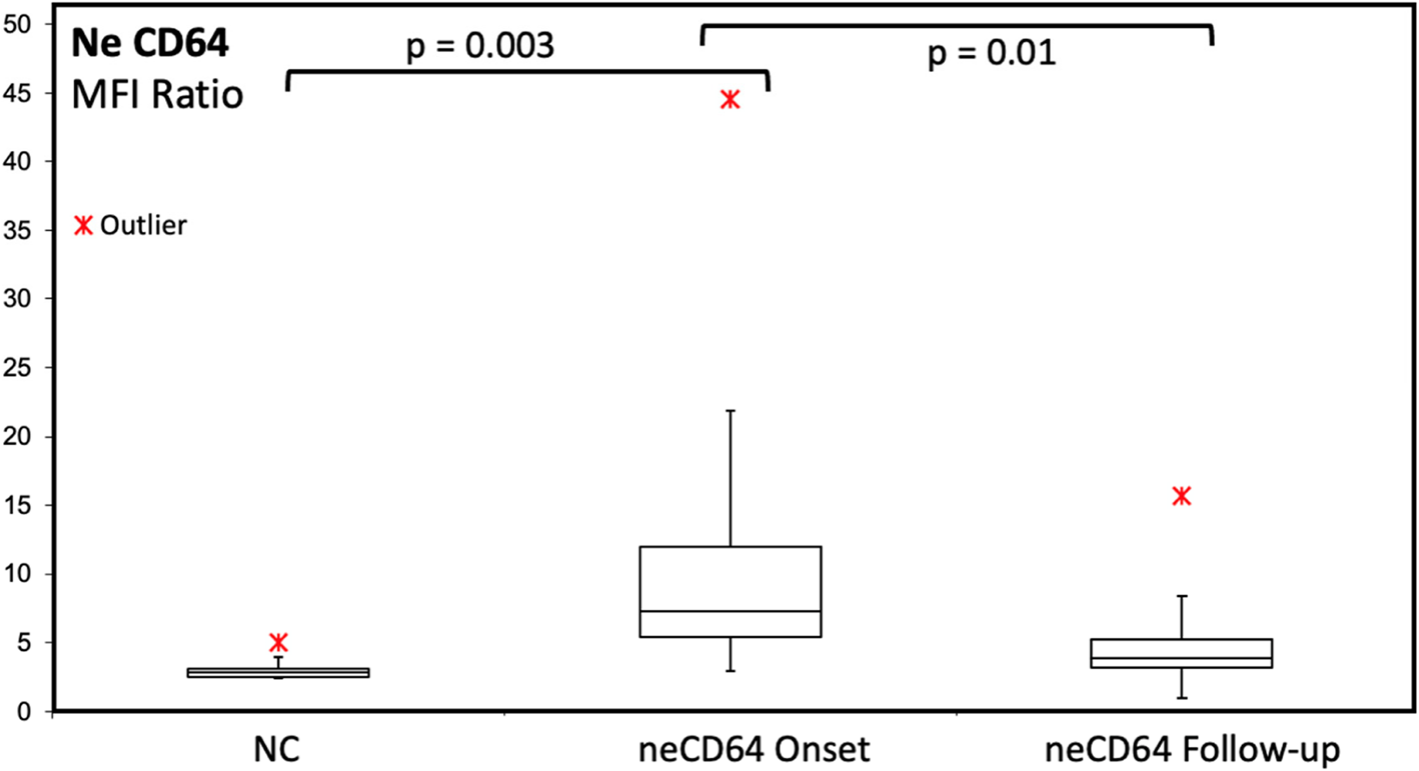
Expression of neutrophil CD64 at disease onset and during the follow-up. A mild upregulation of neCD64 is observed in the majority of patients, while very high values were detectable only in patients with overt bacteremia and sepsis

In addition, during the early disease phase, SARS-CoV2-infected patients showed a slightly increased expression of neCD64, as compared to quiescent granulocytes in the healthy NC group (mean MFI ratio in SARS-CoV2 patients: 10.9 vs 2.7 in NC; *p*= 0.003) (Fig. 2). Nevertheless in most cases, no significant microbiological agents were detected in the culture samples.

**Fig. 3:**
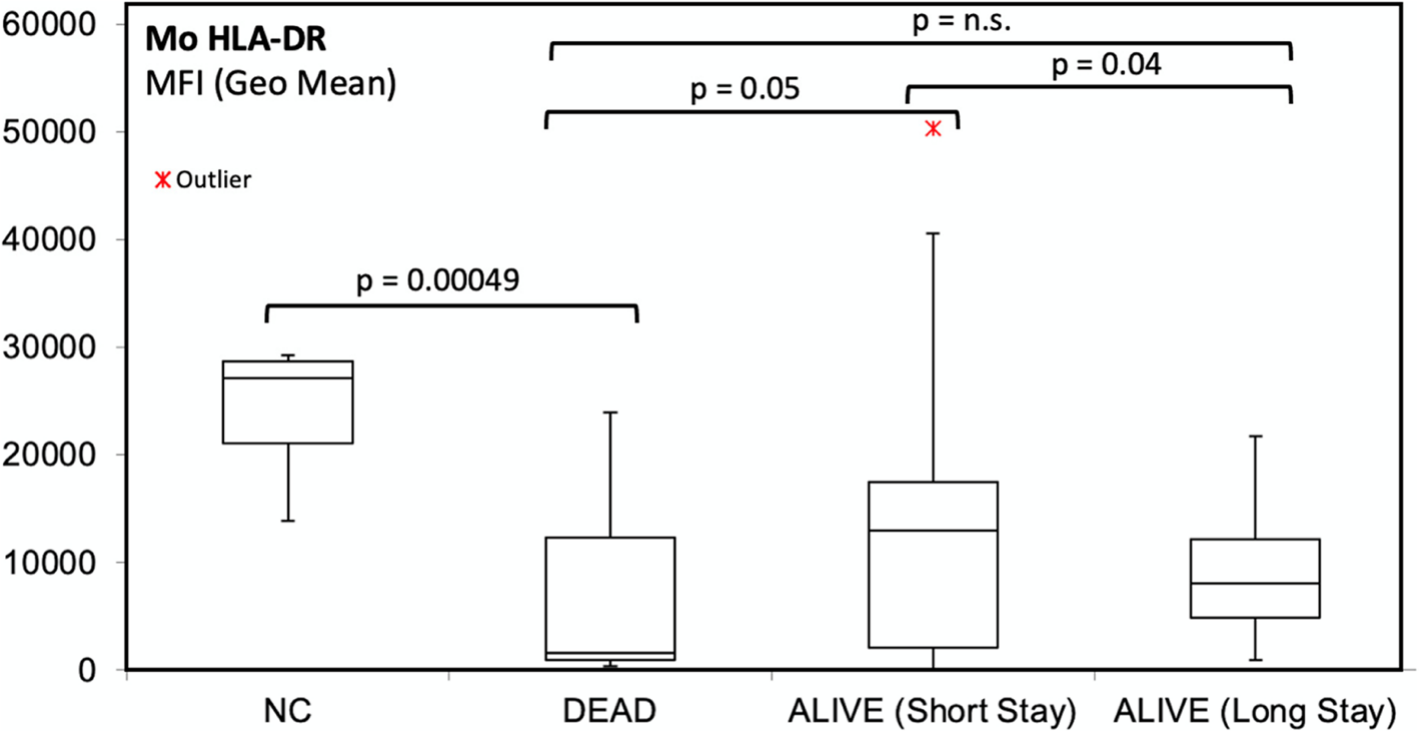
Expression of monocyte HLA-DR as an indicator of immune competence in SARS-CoV2 infected patients. The lowest level of expression was observed in patients who died for multiple complications, whereas patients with prolonged hospital stay showed a lower HLA-DR expression level, as compared with patients with shorter and more favorable clinical course and normal control (NC) subjects

Patients admitted to ICU showed significantly lower values of moHLA-DR than those admitted to other clinical units (mean 7,841 MFI vs 11,754; *p*= 0.017). During the early SARS-CoV2 infection, no significant differences of moHLA-DR expression were found between the six patients who died and those surviving in ICU (mean moHLA-DR expression in ICU patients who died: 6692 MFI vs surviving ICU patients: 9623, p= n.s) (Fig. 3).

Patients with short hospital stay (≤15 days, median 9 days) and good outcome showed higher values of moHLA-DR (17,478 MFI) than long hospital stay patients (>15 days, median 47 days, with mean moHLA-DR MFI: 9590; *p*= 0.04) and than patients who died within 44 days (5437 MFI; *p*= 0.05). No significant differences in moCD169 MFI ratios were found between long and short hospital stay patients.

**Fig. 4:**
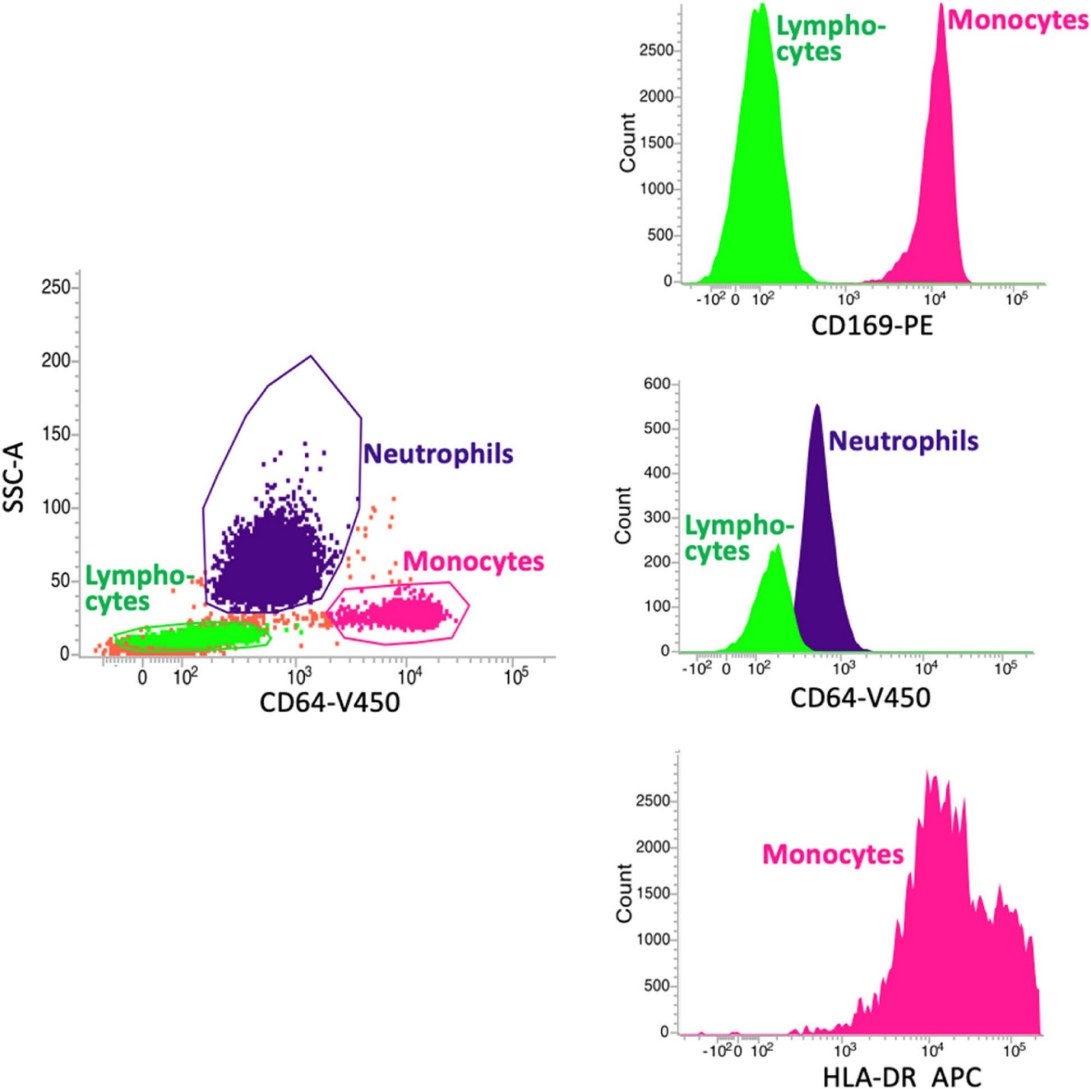
Peripheral blood white cell gating strategy (left panel). Monocytes, neutrophils, and lymphocytes are identified according to their CD64 expression and Side Scatter (SSC-A) features. The diagrams to the right show an example of very high monocyte CD169 upregulation in a patient with active COVID-19, mild neutrophil activation (CD64), and preserved monocyte HLA-DR expression

In most cases, the clearance of the SARS-CoV2 infection-related signs was associated with the normalization of moCD169 within 17 days from disease onset (mean moCD169 MFI ratio returning close to 5). However, in three surviving long hospital stay patients (median 56 days), a persistent upregulation of moCD169 was observed (Fig. 4), with a mean moCD169 MFI ratio =26. In these three cases, the persistent molecular positivity for SARS-CoV2 in upper and lower airway swabs was also detected.

A total of twenty-two patients were admitted to ICU. Nine surviving patients had short hospital stay (<15 days), and 21 surviving patients had long hospital stay (mean 47 days). Six subjects died within 44 days from ICU admission.

**Fig. 5:**
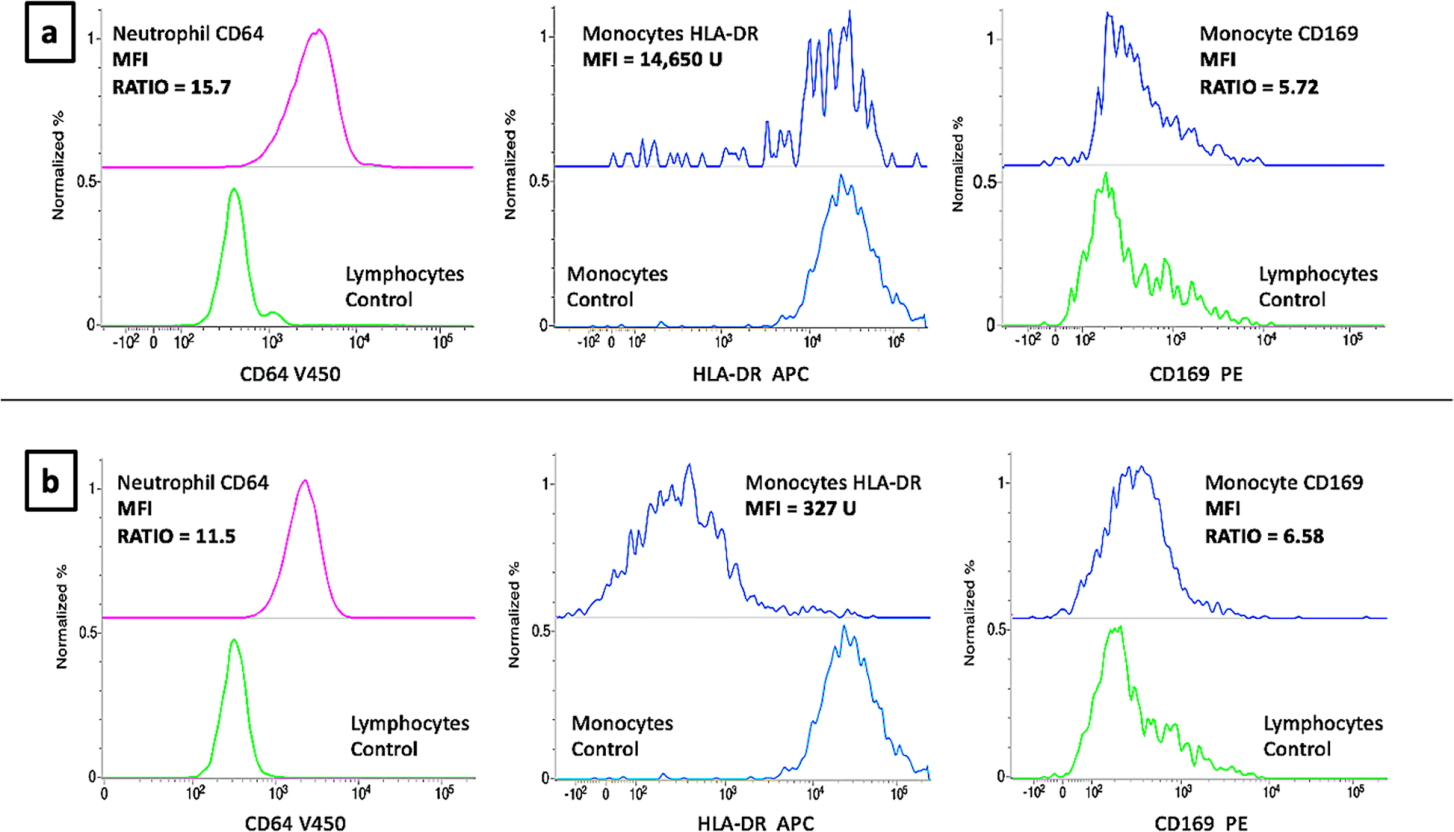
Examples of some of the many possible findings in the monitoring of SARS-CoV2-infected patients undergoing sepsis during their clinical follow-up. Upper row (**a**): Moderate neCD64 upregulation, normal moHLA-DR expression, and cleared SARS-CoV2, with normal moCD169 expression. This patient showed normal immune competence and recovered from sepsis. Lower row (**b**): Moderate neCD64 upregulation, severe moHLA-DR downregulation, and just slightly increased moCD169. This patient died for infectious complications, despite having effectively cleared the SARS-CoV2

During the follow-up, a general reduction of neCD64 expression was found as compared to disease onset (mean MFI ratio from 10.9 to 5; *p*= 0.01). In two cases, a superimposed bacterial sepsis was diagnosed during the hospital follow-up. In one case with neCD64 MFI ratio = 15.7 an overt sepsis was defined with positive blood cultures for *Serratia marcescens.* In the other case with neCD64 MFI ratio = 11.5 and a severely downregulated moHLA-DR MFI, averaging 327, an overt sepsis supported by positive blood cultures for *Staphylococcus capitis* was diagnosed, shortly before the patient’s death (Fig. 5).

In two patients, the progressive and significantly decreased expression of moHLA-DR was associated to fatal outcome in the long term (5834 MFI and 12,320 MFI at onset, respectively, and 2991 MFI and 2865 MFI, respectively, after 10 days from ICU admission). On the other hand, in another ICU patient, the progressive recovery of moHLA-DR was associated to an improving clinical condition that allowed his discharge after 63 days.

## Discussion

The aim of our study was to evaluate retrospectively the changes in the expression of moCD169, moHLA-DR, and neCD64 by flow cytometry in 36 hospitalized patients with severe COVID-19. The marker expression findings with time were correlated to the clinical outcome and the length of hospital stay. In 15 cases, the prospective analysis of these markers with time during the clinical course was also evaluated.

Several studies reported that the presence of risk factors such as obesity, hypertension, cardiovascular, or pulmonary disease might lead to a more severe outcome of SARS-CoV2 infection [25, 26]. In our limited study, we were unable to demonstrate such correlations. Seventeen individuals showed at least three unfavorable comorbidities, but only three of them did not survive. Regarding the other three patients who died, one patient did not show any comorbidity and the remaining two patients showed only two unfavorable comorbidities.

On the other hand and in accordance with other recent studies [27, 28], we found a strong correlation between three flow cytometric indicators with different clinical meaning: moCD169, moHLA-DR, and neCD64 expression and the degree of COVID-19 critical illness, the clinical outcome, and the length of hospital stay.

Patients admitted to ICU showed significantly lower values of moHLA-DR than subjects admitted to other clinical units, indicating that a dysfunctional immune response may contribute to the development of acute respiratory failure [13].

In general, COVID-19 patients in ICU display a more critical and dynamic clinical picture that often gets complicated with time. For this reason, reliable biological indicators that may enable the quick interpretation of the different ongoing signs and symptoms are needed. The flow cytometric monitoring of the combined expression of moCD169, moHLA-DR, and neCD64 seems fitting the purpose.

In a report on a group of 110 patients admitted to the surgical ICU [29], those with a reduced moHLA-DR had a longer median ICU stay (6 days vs. 3 days), independent of the cause of critical illness.

We found that patients with short hospital stay (≤15 days, median 9 days) and good outcome showed higher values of moHLA-DR than long hospital stay patients (>15 days, median 47 days), and than patients who died within 44 days. To our knowledge, these observations were not reported before in patients with SARS-CoV2 infection. The definition of “long hospital stay” was set after 15 days taking into account the changes in viral load. As reported by Doehn et al. [6] between 13 and 15 days since the onset of symptoms, both in mild and severe SARS-CoV2 infection a significant decrease of viral load and moCD169 expression can be detected, along with an increase of serum anti-SARS-CoV2 IgG concentration.

In 33/36 cases, steroid treatment was undertaken along with support therapy in both short hospital stay patients and in ICU long stay patients. The blood testings were performed during the steroid treatment. The steroid administration did not seem however to influence the moHLA-DR expression. As reported by Boomer et al., no significant difference was seen in cytokine production in septic patients treated or not treated with corticosteroids [30].

In the study by Spinetti et al. [13], low moHLA-DR expression remained unchanged within the first 5 days after the ICU admission in critical COVID-19 patients. Conversely, in our 15 cases with a prospective flow cytometric monitoring, the changes of moHLA-DR occurred over a more extended period of time (about 12 days after the first testing) and correlated to the clinical outcome.

It was also reported that moCD169 expression is increased in early SARS-CoV2 infection [5] and returns to normal range within the subsequent 3–4 weeks. CD169 is a type I interferon-inducible receptor; however in the present study, we were unable to evaluate the levels of circulating IFN-gamma.

As reported by other authors we found that in most cases, the clearance of the SARS-CoV2 infection was associated with the downregulation of moCD169 within 17 days from disease onset [6]. However in three surviving long hospital stay patients (mean 56 days), a persistent upregulation of moCD169 was observed, with sustained positivity for molecular SARS-CoV2 swabs. It is reported that severe COVID-19 disease is associated to a high SARS-CoV2 viral load and a longer virus-shedding time [31]. Therefore, a persistent upregulation of moCD169 can be considered as an unfavorable prognostic factor in SARS-CoV2 infection, correlating with a lack of an effective viral clearance.

Therefore, the kinetics of moCD169, moHLA-DR, and neCD64 expression may be useful to discriminate febrile events and to stratify patients according to their risk of complications and length of hospital stay. The quick and real-time characteristic of this flow cytometric assay makes it suitable for on-demand orders with a very short turn-around time (less than 60 min).

Superimposed bacterial infections are the commonest complications in ICU patients. An upregulated neCD64 expression has long been demonstrated as a reliable indicator of severe bacterial infections and sepsis [12]. In our patient cohort, increased values of neCD64 were demonstrated at disease onset, however in most cases without significant positive cultures for bacteria. A significantly low rate of bacteremia was found among COVID-19 patients in the early disease phase [32]. However in another study, the cumulative risk of developing an episode of ICU-acquired bloodstream infection in critical SARS-CoV2 patients significantly increased after 15 days from ICU admission [33]. During our patients’ follow-up, a general reduction of neCD64 expression was observed, although in two cases the neCD64 MFI ratio reached high values in association with positive blood cultures.

Our study has several limitations: the small patient cohort, the initial flow cytometric monitoring not strictly performed at regular intervals during the early phase of the study, and the lost to follow-up of some patients due to their transfer to external ICUs or medical wards during the early SARS-CoV2 epidemic.

## Conclusion

The monocyte CD169 and HLA-DR expression can be used as predictive biomarkers of SARS-CoV2 outcome in acutely infected patients, since they can reliably indicate the status of viral persistence or clearance and the occurrence of superimposed biological phenomena that negatively affect the immune response. The simultaneous assessment of neCD64 expression is of help in discriminating virus-induced fever from severe bacterial infections and sepsis. This real-time flow cytometric assay can be performed on demand, with a short turnaround time (1 h). We believe that the prospective combined monitoring of moCD169, moHLA-DR, and neCD64 can be useful for the management of COVID-19 patients and possibly also for other critical infectious diseases and sepsis.

## Data Availability

Address any request to Dr. Arianna Gatti, corresponding author.
